# Where is information quality lost at clinical level? A mixed-method study on information systems and data quality in three urban Kenyan ANC clinics

**DOI:** 10.3402/gha.v6i0.21424

**Published:** 2013-08-29

**Authors:** Daniel Hahn, Pepela Wanjala, Michael Marx

**Affiliations:** 1 Institute of Public Health, University of Heidelberg, Baden-Württemberg, Germany; 2 Health Information System, Ministry of Health, Nairobi, Kenya

**Keywords:** health information system, clinical information system, data quality, information and communication technology, antenatal care, Kenya

## Abstract

**Background:**

Well-working health information systems are considered vital with the quality of health data ranked of highest importance for decision making at patient care and policy levels. In particular, health facilities play an important role, since they are not only the entry point for the national health information system but also use health data (and primarily) for patient care.

**Design:**

A multiple case study was carried out between March and August 2012 at the antenatal care (ANC) clinics of two private and one public Kenyan hospital to describe clinical information systems and assess the quality of information. The following methods were developed and employed in an iterative process: workplace walkthroughs, structured and in-depth interviews with staff members, and a quantitative assessment of data quality (completeness and accurate transmission of clinical information and reports in ANC). Views of staff and management on the quality of employed information systems, data quality, and influencing factors were captured qualitatively.

**Results:**

Staff rated the quality of information higher in the private hospitals employing computers than in the public hospital which relies on paper forms. Several potential threats to data quality were reported. Limitations in data quality were common at all study sites including wrong test results, missing registers, and inconsistencies in reports. Feedback was seldom on content or quality of reports and usage of data beyond individual patient care was low.

**Conclusions:**

We argue that the limited data quality has to be seen in the broader perspective of the information systems in which it is produced and used. The combination of different methods has proven to be useful for this. To improve the effectiveness and capabilities of these systems, combined measures are needed which include technical and organizational aspects (e.g. regular feedback to health workers) and individual skills and motivation.

Time is running out to achieve the Millennium Development Goals (MDG) by the target year 2015. With 287,000 maternal deaths in 2012, the global situation of maternal health remains unsatisfactory. Unfortunately, the maternal death toll is highest in the African region (1).

Among the established measures to fight maternal mortality is antenatal care (ANC), which is implemented in virtually all countries. However, ANC attendance in the African region is low. Only 74% of all pregnant mothers have at least one consultation with a skilled health worker and only 43% complete a minimum of four visits (global average: 81% and 55%) (1, 2).

To improve maternal health on a global scale effectively and to reach universal health coverage, efforts toward the strengthening of national health systems are inevitable (3). This includes a well-functioning national health information system, which is one of the six health system building blocks suggested by WHO (4–7) .

One of the important subsystems of a fully working national health information system is the routine health information system. It can be described as facilities which report health-related data to the national health information system in regular (mostly monthly) intervals. Data on health outcomes provided by this system are used by decision makers from facility to national level for better, more evidence-based decision making (4).

However, the health information systems in low-income countries are generally known as weak and ‘continue to be plagued by data quality problems’ (4). This causes the situation whereby the low-income countries with high maternal mortality have poorer quality information for effective policy-making and implementation of health programs, for example, Uganda Data Quality Audit (8).

While many of the publications on data quality in low-income countries focus on the higher levels of health information systems, the less-investigated lower levels are of particular importance, since the point of data entry has huge consequences for the data quality of the whole system. If quality of data is hampered from the start, it cannot be restored later on.

However, the role of health facilities in a national health information system is not only the entry point of information. Information reported secondarily is primarily used for clinical purposes (patient care). In this role, a health facility is also a clinical information system in itself: (individual) health information, for example, laboratory results or a patient file in the case of a referral, is essential for the delivery of healthcare and its quality has therefore a huge impact on the service delivery as a whole. The clinical information system has to guarantee appropriate capacities to store, transmit, and process patient data in an appropriate way.

Because of its double function, the health facility holds a key position in the national health information system. Although the importance of health facilities as the entry points of health information systems is acknowledged widely, little research on the heterogeneous clinical information systems (especially in the less regulated private sector) and data quality at facility level in low-income countries has been conducted and published to date (9, 10).

Therefore, this study aims to describe and assess selected clinical and reporting information processes in ANC services of three Kenyan facilities. We applied a multi-methodological approach to assess different aspects of clinical information systems, which are the linkage of individual and aggregate health data, to get a deeper understanding of data flows and points critical for data quality. In addition, we assessed the quality of data from selected exemplary data sources. The combination of individual and aggregate level is a unique feature of this study in resource-poor environments.

## Methods

We employed a multi-method approach to cover different aspects of the information systems and the corresponding data quality. The study design was developed iteratively in cooperation with the study hospitals and the German Agency for International Cooperation (GIZ) in Kenya. We combined different methods to capture a broad picture of the clinical information systems and their capabilities instead of focusing only on (technical) data quality. We argue that the combination of different methods allows not only to assess more aspects of the information systems but also to corroborate the findings which results in more robust results. The whole study took place from March to August 2012 and the researchers were supported by a research assistant from GIZ.

### Selection of study sites

Three hospitals in and around Nairobi were selected for the study. Two private hospitals enrolled in a project supported by GIZ were completed by a purposely selected public hospital to get a wide range of different information systems. All hospitals are level 4 health facilities (6 levels range from 1 for community to 6 for national referral hospitals) and placed in urban areas. Hospital A is a medium-sized hospital and part of an upmarket private network of hospitals and health centers (more than 20 health facilities nationwide). Hospital B is a single private hospital of the same size, offering medium-prized services. On the contrary, Hospital C is a public district hospital. Table 1 gives an overview over selected characteristics of the three study hospitals.

**Table 1:** Selected characteristics of the study sites

	Number of beds	Obstetricians/gynecologists	Midwives/nurses	Annual deliveries	Annual ANC visits	Information system
Hospital A (private)	50	2 (visiting)	5	181	830	M*
Hospital B (private)	52	2 (visiting)	8	168	598	M*
Hospital C (public)	380	3 (2 visiting)	30	11,100	12,000	Mostly P†

* Mixed paper-based and electronic clinical information system

† Paper-based clinical information system.

The hospitals offer full ANC services (as recommended by WHO and the Kenyan Ministries of Health) and maternal care including pre- and postnatal care and comprehensive emergency obstetric care (EmOC, which includes the capability of performing caesarian sections and blood transfusions) (11, 12).

### Workplace walkthroughs

As an entry point to the facilities, informal observations were carried out on five subsequent working days at each study site. Starting from ANC nurses/midwives, all workplaces and members of staff working in or for ANC were visited: nurses/midwives, obstetricians/gynecologists, laboratory technologists, pharmacists, ultrasound radiographers, cashiers, accountants, medical record officers, and data clerks. Special emphasis was put on the generation, transmission, or usage of health information of all kinds. The results of the workplace walkthroughs were used for the selection of the three selected data flows.

### Interviews

Following the workplace walkthroughs, we conducted a series of structured interviews to cover the more subjective dimensions of data quality at the facilities. A score for data quality was developed out of the five dimensions of data quality suggested by the Canadian Institute for Health Information: Accuracy, timeliness, comparability, usability, and relevance – while these dimensions of data quality can differ in names and division, they are included in most of the definitions of data quality. Participants were asked about their opinion on a set of five statements covering the described data quality dimensions. Five-point scales (Likert-type) were used resulting in a maximum data quality score of 25 points (Cronbach's *α*: 0.77).

Sampling was purposeful and included all types of staff taking part in the observations (*n*=44). Classes of staff considered to be more important (mainly nurses/midwives) were weighted by increasing the number of interviewees. Random sampling was considered not useful because of the small numbers of staff working at Hospitals A and B. The interviews were conducted by one researcher at all three hospitals. The structured staff questionnaire was piloted and validated with six members of staff (a gynecologist, nurses/midwives, an ICT technician, a laboratory technician, and an accountant) at one hospital.

Key informant interviews with five members of management at each facility completed our methodology (*n*=15). The head of facility (CEO/medical superintendent), chief gynecologist/obstetrician, matron, head of ICT department, and chief accountant provided information on structural data of the hospitals, the managements’ view on data quality, reporting, and data usage at the study sites.

### Assessment of data completeness and accuracy

Finally, we selected three information processes for an assessment of data completeness and accuracy in ANC at the three study sites. Accuracy is the percentage of correctly transmitted items from the original data source in secondary data sources.

Two processes contain individual patient health data and one is the preparation and transmission of monthly ANC reports. The applied method gives an impression of the hospital's information system's capacity to transmit patient information completely and accurately. Every workplace where data are used, processed, or transmitted has to be considered as a potential risk to data quality.

For the two processes at patient level, a set of three data tracer items each was chosen and for the process at reporting level, a set of five data tracer items was chosen. Table 2 gives a comprehensive overview over the selected data items and the data sources in which they were followed up. The sources are ordered chronologically by entry time.

**Table 2:** Description of the data tracer items used for the assessment of data completeness and accuracy

Data tracer set	Data tracer items	Followed up in
Patient level		
Laboratory data sample	Blood group, Rhesus factor, hemoglobin level	*Laboratory register* (LT), electronic medical record (LT), Mother and Child Health Booklet (AN), ANC Register (AN)
ANC nurse data sample	Systolic and diastolic blood pressure, weight	*Mother and Child Health Booklet* (AN), electronic medical record (AN), ANC register (AN)
Record level		
ANC report data sample (monthly)	ANC clients, new ANC clients, ANC revisit clients, ANC 4th visit clients, clients with Hb < 7 g/dl	*ANC register* (AN), MOH 711 report form (AN), national health information database (AN)

Data source (person responsible for data entry): LT, laboratory technician; AN, ANC nurse. Reporting with support from Medical Records Department. Original source is shown in italic. Laboratory and ANC nurse data sample are part of the routine clinical information. ANC report data sample is part of the monthly routine reporting.

The data sets were chosen because of their high clinical relevance and also practical reasons. The first one was selected from laboratory data (called laboratory data sample) and contains blood group, Rhesus factor, and hemoglobin level as tracer data items. They are originally recorded by laboratory technicians in the laboratory register. The tests are performed at the client's first ANC visit (among others). The second data set (ANC nurse data sample) consists of vital signs (systolic and diastolic blood pressure, and weight). They are recorded by the ANC nurses/midwives in the Mother and Child Health Booklet (Ministry of Health document number 216/MOH 216). This booklet is compulsory by Kenyan regulations and the main (clinical) data source used at all study sites. Its integration in the assessment of data quality is a special feature of our methodology since most of the data quality audits carried out start at register level only.

The Health Booklets of the clients of two subsequent days were used for the assessment. One week later and using the clients’ full names, the tracer data items were followed up in the ANC Register (Ministry of Health document number 405/MOH 405) and – if available – the electronic medical record system. Clients who did not undergo blood tests at the study site were excluded for the laboratory data sample.

For the third data set, a slightly different approach was used for the assessment because the tracer data items in the monthly reporting process are aggregate data. They are processed by a nurse/midwife through manual summarization and the original data source is the ANC Register (MOH 405). Up to 23 monthly indicators are entered in various external and internal reports. Out of these, five tracer items were selected and followed up for 3 months (April–June 2012) retrospectively (Table 2). The time frame was severely limited by the low availability of older reports.

Independent double-counting and processing of the indicators was used to create a gold standard for the tracer data items. If discrepancies occurred, the item was recounted manually by a researcher. In a second step, the gold standard indicators were compared with the reports prepared at the study sites.

### Data processing and statistical testing

All quantitative data were entered in separate computer databases (institutional records and staff interviews) using interfaces developed with Microsoft Access 2010 (Microsoft Corporation, Redmond, USA). Double entry technique was used to create a gold-standard for data quality. Differing data items were manually reviewed by the researchers. For statistical testing, all sets of data were converted into SPSS Statistics version 21 (IBM, Armonk, USA).

Data were analyzed with descriptive statistics. Additionally, we used Kruskal–Wallis test for three groups and Mann–Whitney *U* test for two groups to compare relevant continuous measures (especially scores).

Qualitative data (staff survey and key informant interviews) was processed manually. Answers were classified and grouped by facility and staff class.

### Limitations

In addition to the general methodological limitations of multiple case studies (small number of study sites and purposeful sampling), the study was limited by the low availability of data sources suitable for the assessment of data quality.

### Ethics statement

Informed consent was gathered from all participants of the study. The method used for consent taking depended on the study method. For the workplace walkthroughs and interviews, verbal consent was given by the participants and documented by signature. Verbal consent without documentation was used for patients participating in the workplace walk-throughs and for the usage of medical records and reports, consent was given by the head of the study hospital on behalf of the patients.

The protocol was approved by the Institutional Research and Ethics Committee of the Moi University, College of Health Sciences, Kenya (IREC 000847) and the Ethical Board of the University of Heidelberg, Germany (S-050/2012).

## Results

### Patient flow in ANC and clinical information system

The patient flow was highly standardized and the basic structure was very similar at the three study sites: an ANC visit started with the client being registered at the front desk before meeting a nurse/midwife at the ANC department. Once there, vital signs were taken (represented in our assessment by the ANC nurse data sample) and recorded in the client's Mother and Child Health Booklet (MOH 216). Then, the client would be sent to the laboratory (facultative, but regularly at the first ANC visit) and results were recorded in the laboratories’ registers first (represented by Laboratory data sample). Also ultrasound scans were done at all study sites, but they were seldom performed at Hospital C.

After all diagnostics were conducted, the client consulted an obstetrician (at Hospitals A and B) or an ANC nurse/midwife at Hospital C, where obstetricians were consulted only in the case of complications. The results of laboratory tests were copied to the Mother and Child Health Booklet by a nurse/midwife. Later, a set of 36 patient details and results was copied to the ANC register. This was done by a nurse/midwife again. Finally, the client could be sent to the pharmacy to collect prescribed drugs.

Billing had high priority and caused high workload at Hospitals A and B, since every single service was billed separately there. If a patient was cash-paying, all services had to be paid at the cashier's office first. Clients with a confirmed valid insurance cover did not have to pay at the facility; the services were billed later by the hospital.

Though the patient flows were very similar at the study sites, the time clients spent at the hospital for one ANC visit ranged from 30 minutes to few hours at the private Hospitals A and B up to 2 days at the public Hospital C. At this hospital, a fully paper-based system was used for information exchange and Hospitals A and B had implemented additional electronic clinical information systems. Apart from billing and accounting, the systems were supposed to be capable of medical records and also the transmission of laboratory results from laboratory to the clinicians.

While Hospital A used these functionalities fully, Hospital B did not use medical record functions for ANC. Against the findings from the interviews, a test of the system by the researchers showed that it did not include medical records for ANC clients. To bridge this information gap, Hospital B kept the Mother and Child Health Booklet at the facility. Also, laboratory results could not be searched for by clients’ names resulting in inaccessibility of laboratory results (which were copied into the system, but not accessible).

### Reporting in ANC

Reporting of workload and patients was performed by nurses/midwives at all three facilities. Nurses were supposed to count patients and characteristics in the ANC register and transfer the aggregate data to the National integrated form for reproductive health, HIV/AIDS, malaria, TB, and child nutrition (Ministry of Health Form Number 711a). Though the use of a register was compulsory, it was only used at Hospitals A and C, leaving the Mother and Child Health Booklets as only source of information at Hospital B.

From the reporting form, the information was copied to the District Health Information System 2 (DHIS 2) using a web-based online entry form. The DHIS 2 is Kenya's electronic health information system and currently under implementation. Later, the (paper form) MOH 711a was sent to the District Health Records and Information Officer.

**Fig. 1:**
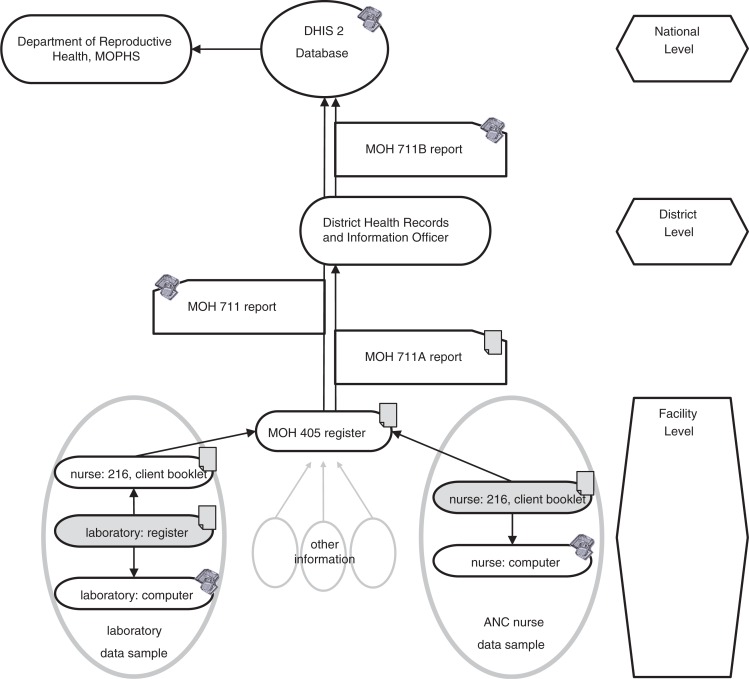
Selected information flows in clinical communication and reporting in the ANC departments. Original information sources are highlighted in grey.

A third report for internal purposes, which differed in size and range, was written and used at all three facilities. The information flows are illustrated in Fig. 1.

### Data quality from staff's perspective

Using the data quality score, staff at the ICT-supported hospitals A and B rated the quality of patient-level data significantly higher (20.8, SD 1.77 and 21.7, SD 2.86; *p*<0.001; maximum of 25 points) than staff at Hospital C (17.1; SD 3.14). In the subgroup clinical staff (clinicians and nurses/midwives), differences were even slightly higher (20.9, SD 1.78 and 21.2, SD 2.86 vs. 16.3, SD 3.14; *p*=0.007). We found no significant differences between the dimensions of data quality.

At all study sites, health workers complained about delayed laboratory results, but at Hospitals A and B delays reported were within minutes or hours and at Hospital C up to 24 hours and longer. At this facility, delays occurred also because patients tended to forget the Mother and Child Health Booklet (MOH 216). This did not happen at Hospital A (electronic medical record system) and Hospital B (where the booklets are kept at the facility).

Missing information in reports and at individual level was a common concern of health workers at all study sites and at Facility C also underreporting of patients at facility level was a known phenomenon. This probably occurred rather rarely at the private hospitals A and B since proper documentation was needed for billing and accounting.

Members of staff also mentioned limited accessibility of medical records frequently. On the one hand, health workers (mostly at Hospital C) found it challenging to retrieve old (paper) patient files and that they had no backup system for a lost Mother and Child Health Booklet – The ANC register was not used for clinical purposes because this was found impractical. On the other hand, health workers at Hospitals A and B complained about poor digital user rights management leaving them without access to needed medical records.

Health workers reported that information was particularly often lost in the case of emergency referrals. Especially (but not only) at Hospital C, referrals without proper documentation were a common problem. Reasons could be found in weak data management and unsatisfying standardization of referral forms, but also patients could hide their test results, as a midwife at Facility B explained: ‘A mother told me that she had forgotten the booklet, but after the delivery I saw the booklet in her bag. When I checked it, I found her to be HIV seropositive. She was afraid that I would not conduct the delivery’.

### Feedback from staff's perspective and usage of (aggregate) data at the study sites

The majority of interviewees were involved in reporting: The number was higher at Hospital C (12 out of 15) than at Hospitals A and B (9 out of 15 and 9 out of 14). However, the majority of involved staff reported that they had never received any kind of feedback, especially at Hospitals A and C. At Hospital B, feedback was more common (8 out of 14) where it was mostly comments on performance and was usually given by a supervisor on a monthly base. Only four members of staff at Hospital C (and one at Hospital B) reported to have ever received extensive feedback including comments on information quality.

The usage of (aggregate) data was not very common with the exception of Hospital B which used data for monitoring (mostly clinicians). However, data were used a lot for one specific purpose: information processing and usage in finance and accounting (at the private hospitals A and B). Data were also used for long-term business decisions. As reduced data quality could result in financial losses, the hospitals undertook huge efforts to increase the quality of financial data. However, the positive effects were limited to financial data.

### Completeness and accuracy in ANC health records

In total, the Mother and Child Health Booklets (MOH 216) of 92 clients were included in the assessment and out of them, 15 were excluded for the laboratory data sample, because they had received their blood tests at another facility.

Completeness of patient level data was slightly limited in almost all clinical documents (Table 3) and several data sources showed structural weaknesses. First, the laboratory registers at Hospitals A and B consisted only of (more or less sorted) loose papers and though the researchers had undertaken efforts to find all documents, only 71.7% (43/60, Hospital A) and 77.8% (56/72, Hospital B) of tracer items could be retrieved. Documents not found were considered as lost. Completeness was much higher at Hospital C (96.7%, 96/99) where a register book was used, but also in this register one patient was missing. Second, Hospital B used a medical record system without patient file functionalities: laboratory results were not available for health workers 24 hours after the laboratory had been conducted. Therefore, we extracted laboratory data manually from the database resulting in higher completeness rates (88.9%, 64/72) than in the laboratory records (77.8%, 56/72).

**Table 3:** Completeness of data tracer items in clinical routine communication in ANC

	Hospital A (private)	Hospital B (private)	Hospital C (public)
*Laboratory data sample in …*			
… laboratory register	71.7% (43/60)	77.8% (56/72)	96.7% (96/99)
… electronic medical record	96.7% (58/60)	88.9% (64/72)	No CIS
… Health Booklet	100% (60/60)	90.3% (63/72)	96.7% (96/99)
… ANC Register	85.0% (17/20)	No MOH 405	42.4% (14/33)
*ANC nurse data sample in* …			
… Health Booklet	95.8% (69/72)	100% (99/99)	100% (105/105)
… electronic medical record	86.1% (62/72)	Not saved	No CIS
… ANC Register	97.2% (70/72)	No MOH 405	91.4% (96/105)*

* Three patients missing completely.

Three data items per patient per source with the exception of laboratory data in the ANC register, since only Hb level is recorded there.

However, completeness of laboratory data in the Mother and Child Health Booklet (MOH 216) was good ranging from 90.3% (Hospital B) to 100% (Hospital A). Observations had shown that paper backups were used for all data transmission at Hospitals A and B. This could offer an explanation to the higher completeness rates in the booklet compared to the Clinical Information System at Hospital A and especially at Hospital B.

Hemoglobin level was copied to the ANC register only rarely (85%, 17/20 at Hospital A and 42%, 14/33 at Hospital C). At facility B, the compulsory ANC Register (MOH 405) was not used. If a patient used the Mother and Child Booklet as intended and kept it, the hospital was left without any records.

Completeness of the ANC nurse data sample in the Mother and Child Health Booklet was higher and ranged from 95.8% (69/72, Hospital A) to 100% (99/99 and 105/105 at Hospitals B and C). However, three patients were missing in the ANC register at facility C which resulted in underreporting by around 10%. And since Hospital B did not use registers, an assessment was not possible at all.

When looking at the accuracy of data, figures changed slightly because only available data was audited. Comparing existing data items at different data sources showed better results only at the first view. Despite the use of ICT support, with 90.2% (37/41) and 89.8% (44/49) accuracy levels were found to be lower at the private hospitals (A and B) than at the public Hospital C with 95.7% (89/93). This is of particular importance because it included several ‘switches’ of blood group: This was the case for two patients each at Hospitals A and B and for one patient at Hospital C. Additionally, two changes in Rhesus factor occurred at Hospital C (one from negative to positive and one from positive to negative).

Accuracy was also limited in the ANC register for the ANC nurse data sample (82.1%, 55/67 at Hospital A and 87.5%, 84/96 at Hospital C, Table 4).

**Table 4:** Accuracy of transmission of tracer data items in clinical routine communication in ANC

	Hospital A (private)	Hospital B (private)	Hospital C (public)
*Laboratory data sample*			
Laboratory >>X*>> booklet	90.2% (37/41)	89.8% (44/49)	95.7% (89/93)
(Hb only) booklet >> MOH 405	88.2% (15/17)	No MOH 405	85.7% (12/14)
*ANC nurse data sample*			
Booklet >> MOH 405	82.1% (55/67)	No MOH 405	87.5% (84/96)
Booklet >> computer	91.7% (55/60)	Not entered	No EMR

* At Hospitals A and B, the information is transferred electronically, at Hospital C with a (paper) report form. However, at all study sites, it is finally copied to the Health Booklet.

Accuracy of transmission of tracer data items from one clinical data source to another. Again, Hb level is the only laboratory data item transferred to the ANC register.

### Completeness and accuracy in ANC monthly reports

We found limitations of data quality in the reports, too. Completeness of reports was mixed, ranging from poor 60% (27/45) at Hospital B to good 97.8% (44/45) at Hospital C (Table 5). However, accuracy was more severely reduced: even with a deviation of 5%, only 22.2% (6/27) at Hospital A and 55.3% (21/38) tracer data items matched with our gold-standard, calculated from the ANC Register (MOH 405). Even worse, at Hospital B the assessment was not conducted since no register was used.

**Table 5:** Completeness and accuracy in several (monthly) maternity reports (including ANC)

	Hospital A (private)	Hospital B (private)	Hospital C (public)
Completeness ANC	86.7% (39/45)	60.0% (27/45)	97.8% (44/45)
Accuracy ANC	14.8% (4/27)		5.3% (2/38)
Accuracy ANC (with 5% deviation)	22.2% (6/27)		55.3% (21/38)
Deviation ANC fourth visit, monthly at average	800.0%*		53.06%†
Continuity of reports	60.0% (9/15)	95.8% (23/24)	83.9% (26/31)

* Over-reporting

† mixed over- and under-reporting.

Continuity describes the consistency of the reports which must not be mixed with actual accuracy which is measured against a gold-standard created by the researchers.

If only exactly matching figures were counted, data accuracy was even lower: while at Hospital A still 14.8% (4/27) data items were found to be correct, it was only 5.3% (2/38) items at Hospital C.

Therefore, we tried to compare the continuity of the three reports at the study sites as a measure for data quality. We created an indicator by comparing the data items in the three reports in pairs and counting the matching pairs. We found deviations even between the different reports, created from the same data source. Paradoxically, with 23 out of 24 pairs matching (95.8%), Hospital B was found to have the highest continuity in ANC reports but this did not necessarily mirror higher accuracy of the reports.

Also the deviations of the single data items were severe. As an example, the indicator ‘Number of clients completed 4th Antenatal visit’ (Report form MOH 711a) has practical relevance as a measure of completion of the WHO-suggested course of focused ANC. However, with average monthly deviations of up to 800% at Hospital A and 53.1% at Hospital C, its practical relevance as a measure might have to be challenged.

## Discussion

Data production for the national health information system starts with patient–provider interactions at facility level. Investigating the quality of the information systems at the facilities was found to be challenging, since a clear definition is missing and very different aspects had to be included. Combining different methods and viewing the information systems from different viewpoints showed additional benefits compared to a merely technical assessment of the quality of data sources. Not only more dimensions of the capabilities and the quality of the information systems were covered but also suggestions for the improvement of data quality can be drawn from our qualitative results. An additional benefit might be that the combination of methods is also likely to produce robust results in other settings, since the quality of record keeping can easily be too low for a more formal assessment (especially if data sources are missing completely).

### Data quality

High quality of data is vital for patient care and (as aggregate data) also needed for evidence-based decision making at the higher levels of the Kenyan health system. However, the examined systems at facility level were only partly adequate and data quality was rather limited. The limitations appeared in individual patient health records as well as in aggregated reports and agree with the results of other researchers and also the World Health Organization (4, 13–16). But again, there still seems to be a huge and unmet need for research in this area.

Health workers and other members of staff rated individual patient health data quality higher at the two private hospitals than at the public hospital (20.9 and 21.7 out of 25 vs. 17.1), but our assessment of ANC health records did not show better completeness and accuracy at the private (and ICT-supported) hospitals. The high number of wrongly transmitted laboratory results is not acceptable and it is noteworthy that most of the respondents were unaware of the limited data quality.

We also found all examined reports of limited completeness, accuracy, and even continuity. The compulsory ANC register was not used at one private facility. But even where it was used, the accuracy of the reported figures was as low as 5.3% at Hospital C and only 14.8% at Hospital A. Manual processing might be a reason for the reduced accuracy at Hospital C, but also the semi-manual preparation of reports (with Microsoft Excel™) at Hospital A showed still unsatisfying (but better) results.

### Technical problems and solutions

A technically well-designed set of data management tools (for records and reports) forms the base of every information system (17). We found well-designed standardized Health Booklets for clinical use in ANC and also the report forms were designed in an easy to use way.

However, reporting was said to be very time-consuming and the computerization of reporting processes could offer help. Even more, ICT has shown positive effects on data quality and even quality of care in a number of research settings in low-income countries (18–21). In our study, health workers were more satisfied with data quality at their workplace if they worked with a computer (e.g. a faster transmission of laboratory results was mentioned frequently).

But contrary to the positive attitude toward ICT expressed by virtually all respondents, we did not find a linkage of completeness and accuracy of data and the use of information and communication technology. These findings support the doubts of many researchers that a merely technical approach toward data quality is not sufficient for improvement (4, 17). However, thorough evaluation of electronic clinical information systems and their impact (especially for commercially available and widely used ones) is missing (17).

So while the proof of effectiveness for information and communication technology is still absent, it is implemented as a solution for a bench of different problems including data quality. But it has to be kept in mind that ICT adds other challenges and poorly designed or adapted systems can be a threat for data quality in itself. For example, the impossibility of accessing laboratory data at Hospital C and missing laboratory results in the computer system at Hospitals A and C decreased the quality of information significantly. Including electronic clinical information systems in the regulation of therapeutic goods might be helpful.

### The individual health worker at the ground

As Lafond et al. point out, the capability of the clinical information systems is also influenced greatly by the individual skills and behavior of the staff (17) and, therefore, individual data management skills should be strengthened. As an example, the high deviations of the indicator ‘Number of clients completed 4th Antenatal visit’ (Report Form MOH 711) might be caused by a misunderstanding; while some health workers seemed to report the number of mothers completing a course of four ANC visits, others reported the number of mothers with four or more visits.

Furthermore, health workers rather prioritize patient care instead of data management and reporting duties (17). While this might explain the limited quality of aggregate data in the reports, it cannot explain the wrongly transmitted laboratory results. As they have a direct negative influence on patient care, it can be supposed that they are at the core of health workers’ interests toward patients. We therefore interpret that awareness of data quality is generally low and that the errors in the transmission of laboratory results were simply not known and therefore not addressed (while at the same time data quality was rated good or very good). Technical assistance like automated validity checks at data entry might be a supporting but not sufficient method to improve the quality of information. It probably has to be accompanied by a general process of sensitization for data quality.

However, not everything is about individual skills. At national level, there is also a huge gap of professional data managers at district and especially at facility level. This problem was not to be found at the study sites.

### Integrated health information systems

Also more structural and even cultural aspects influence the capability of the information systems. When it comes to reporting, we found the typical ‘one-way’ reporting system which is endemic in many low-income countries (4). It is characterized by the imbalance of nurses/midwives (who are already overburdened with clinical duties) recording information and higher levels of the health system using this information. Even feedback on the reports was rather seldom. Of course, this can have a negative impact on health worker's motivation to take part in reporting processes (17) as the matron of Hospital C pointed out: ‘People are not aware what data is needed for. So reporting seems a bit useless and more insight would raise the level of motivation’. Not much is known about the motivation of health workers. While single interventions like incentives for higher data quality have shown positive effects (13), more research of the motivation of health professionals and other potential data users and their attitudes toward data quality (and usage) is needed.

The described imbalance in reporting has a more structural dimension forming also a barrier to increased data usage at facility level: most of the ANC indicators are output or outcome indicators. Designed for program monitoring and evaluation, they are of relatively little use at facility level. Therefore, an additional focus on process indicators might not only have a positive impact on data quality management at facility level but also increase the motivation for data processing (15, 22, 23).

At the study sites, data quality was limited by many different factors. In conclusion, a systematic intervention with a wide range of actions (including technical, individual, and organizational aspects) is needed to transform the found rather one-way systems into an integrated health system with higher data quality and to promote a culture of information usage (17, 24). While increased data utilization would be at the core of this intervention and work as a motor for change, it is not a guarantee for higher data quality. Also the existing system of feedback and supervision from higher levels of the health system should be strengthened (not only) to ensure the (correct) usage of compulsory documents, for example, through periodic random supervisory checks at the health facilities. The strengthening could also include the extension of institutionalized data quality feedback workshops (as currently successfully performed in Kenya at provincial level) to facility level (5). In these meetings, not only feedback on quality of data is given, but also a self-assessment of data quality and processing is performed and finally critiqued by colleagues to increase the level of quality awareness and data usage.
